# The Impact of Exercise on Overall Survival in Patients With Metastatic Colorectal Cancer: A Systematic Review and Meta-Analysis

**DOI:** 10.7759/cureus.97682

**Published:** 2025-11-24

**Authors:** Ryuichi Ohta, Taichi Fujimori, Kaoru Tanaka, Chiaki Sano, Hidetoshi Hayashi

**Affiliations:** 1 Community Medicine, Unnan City Hospital, Unnan, JPN; 2 Medical Oncology, Kindai University Faculty of Medicine, Osaka-Sayama, JPN; 3 Community Medicine, Shimane University Faculty of Medicine, Izumo, JPN

**Keywords:** exercise, meta-analysis, metastatic colorectal cancer, overall survival, physical activity, systematic review

## Abstract

Although physical activity is known to improve outcomes in non-metastatic colorectal cancer (CRC), its effect on metastatic colorectal cancer (mCRC) remains unclear. This study systematically reviewed and meta-analyzed the association between exercise and overall survival (OS) among patients with mCRC. A comprehensive search of PubMed, Embase, and Web of Science was conducted for studies published from 2000 to September 2025. Eligible studies evaluated exercise or physical activity in adults with histologically confirmed mCRC and reported survival outcomes. Hazard ratios (HRs) for OS were pooled using a random-effects model. Study quality was assessed using the Newcastle-Ottawa Scale (NOS), and heterogeneity was evaluated with the I² statistic. Four prospective cohort studies comprising 2020 patients met the inclusion criteria. Exercise types were predominantly mild to moderate aerobic activities such as walking or light household chores. The pooled HR for OS was 0.64 (95% CI, 0.48-0.87), indicating a 36% reduction in mortality among physically active patients compared with those who were inactive. Heterogeneity was substantial (I²=74.5%), but the direction of effect was consistent across studies. All studies were of moderate to high quality (NOS 7-8). Funnel plot analysis showed mild asymmetry, suggesting possible publication bias. Mild to moderate physical activity is significantly associated with improved OS in patients with mCRC. Even low-intensity activities such as walking may offer survival benefits and are feasible for patients with advanced disease. These findings support the incorporation of moderate exercise into multidisciplinary care for mCRC, although the observational nature of the available studies limits the ability to draw causal inferences. Large-scale prospective trials are warranted to determine optimal exercise regimens in this population.

## Introduction and background

Colorectal cancer (CRC) is one of the most common malignancies worldwide, with approximately 1.9 million new cases and 930000 deaths reported in 2020 [1,2]. It ranks second globally in both incidence and mortality, representing a significant public health challenge [3]. In Japan, the incidence of CRC continues to rise, with approximately 150000 new cases and 50000 deaths estimated in 2022.

While early-stage CRC can often be managed successfully through surgical resection, achieving five-year survival rates of 70%-90%, the prognosis for patients with metastatic colorectal cancer (mCRC) remains poor, with a five-year survival rate of approximately 15% [4]. Despite advances in systemic chemotherapy, molecularly targeted agents, and immunotherapy, median overall survival (OS) has improved by only approximately 30 months, and a cure remains elusive [3,5].

In recent years, cancer care has increasingly emphasized not only prolonged survival but also preserving patients’ quality of life (QOL) and functional capacity, including activities of daily living (ADL) [6]. For individuals with mCRC, these concerns are particularly salient because systemic therapy frequently induces fatigue, sarcopenia, and functional decline, all of which diminish activities of daily living and overall well-being [6]. Among supportive strategies, lifestyle modification, including diet and exercise, has gained attention for its potential to enhance systemic health, treatment tolerance, and overall prognosis. Studies in early and locally advanced CRC have shown that patients engaging in regular physical activity exhibit lower recurrence and mortality rates [7]. Additionally, randomized controlled trials in postoperative CRC patients have demonstrated that structured aerobic or resistance exercise can improve physical function, reduce fatigue, and enhance QOL [8].

However, evidence regarding the role of exercise in patients with metastatic disease remains limited. For individuals with mCRC, treatment-related fatigue, sarcopenia, and declining performance status often restrict the ability to engage in exercise [7,8]. Existing reviews have examined mainly physical activity in non-metastatic or mixed-stage CRC, and evidence specific to mCRC remains limited. To date, these metastatic-specific associations have not been systematically consolidated with a focus on survival outcomes, underscoring the need for a dedicated synthesis in this population.

Therefore, the present study aimed to systematically review and meta-analyze the relationship between exercise and clinical outcomes, particularly OS, among patients with mCRC. By integrating available evidence, this study aims to clarify whether exercise confers a survival benefit and to support its potential inclusion as a key component in multidisciplinary care for patients with mCRC.

## Review

Study design

This study was conducted as a systematic review of observational cohort studies according to the PRISMA 2020 guidelines for reporting systematic reviews and was prospectively registered with the International Prospective Register of Systematic Reviews (PROSPERO; registration ID: CRD420251164894) [9].

Eligibility criteria

Studies were eligible if they evaluated the association between exercise and clinical outcomes in patients diagnosed with mCRC. Both observational and interventional studies were considered, provided that sufficient quantitative data were available to estimate hazard ratios (HRs) or relative risks for OS. The study selection process was conducted independently by two reviewers (R.O. and T.F.), and discrepancies were resolved through discussion or consultation with a third reviewer.

Inclusion Criteria

Studies were included if they met all of the following criteria: adult patients with histologically confirmed mCRC; assessment of exercise or physical activity, whether structured or self-reported; inclusion of a comparison group with lower or no activity; reporting of OS, treatment-related outcomes, or quality-of-life measures; prospective or retrospective cohort design, case-control design, or secondary analysis of clinical trials; and publication as an original peer-reviewed article in English.

Exclusion Criteria

Studies were excluded if they met any of the following conditions: populations limited to non-metastatic CRC or studies not clearly differentiating metastatic disease; absence of physical activity or exercise as an exposure; publication types such as case reports, reviews, editorials, commentaries, or abstracts without full text; insufficient data for estimating HRs or equivalent outcomes; pediatric or adolescent populations; and non-English or non-peer-reviewed sources.

Data sources and search strategy

A comprehensive literature search was conducted using the PubMed, Embase, and Web of Science databases. Additional searches were considered in the Cochrane Library and Scopus to ensure thoroughness. The search strategy used both Medical Subject Headings (MeSH) and free-text terms related to colorectal cancer, metastasis, and exercise. The following core search string was applied "(“Colorectal Neoplasms” OR “Colorectal Cancer” OR “Colon Cancer” OR “Rectal Cancer” OR “colorectal carcinoma”) AND (“Neoplasm Metastasis” OR metastasis OR advanced OR “stage IV”) AND (“Exercise” OR “Exercise Therapy” OR “Physical Fitness” OR “Exercise Movement Techniques” OR exercise OR “physical activity” OR “resistance training” OR aerobic OR “strength training” OR rehabilitation) AND (“Quality of Life” OR “quality of life” OR QOL OR fatigue OR “physical function” OR “muscle strength” OR endurance OR survival OR “treatment outcome”[Mesh] OR biomarker OR “immune marker” OR adverse event OR toxicity)."

The search covered studies published from January 2000 to September 2025, reflecting the era of modern systemic treatment for mCRC. Only English-language articles were included to ensure consistency and quality. Grey literature, such as conference abstracts or unpublished studies, was excluded due to a lack of peer review. To ensure reproducibility, the full search strategies, dates, and the number of retrieved records were documented and will be provided upon request.

Study selection

Two independent reviewers screened titles and abstracts for relevance, followed by a full-text review of potentially eligible articles. A third reviewer resolved discrepancies. The final selection was documented using a PRISMA flowchart.

Data extraction

Data extraction was performed independently by two reviewers using a standardized, pre-designed data collection form, in accordance with the PRISMA 2020 guidelines [9]. From each eligible study, the following information was systematically collected: study characteristics (first author, publication year, country, and study design); patient characteristics (sample size, median or mean age, sex distribution, and performance status (e.g., Eastern Cooperative Oncology Group (ECOG 0-1)); exercise characteristics (type (aerobic, resistance, or combined), intensity, frequency, and duration); and OS, when available.

When HRs and 95% confidence intervals (CIs) were reported, data were directly extracted. If not explicitly provided, HRs were calculated from Kaplan-Meier curves.

The methodological quality of included studies was assessed using the Newcastle-Ottawa Scale (NOS) for observational studies [10]. The NOS evaluates three domains, selection, comparability, and outcome, each with a maximum of three points. Discrepancies between reviewers were resolved through consensus or consultation with a third reviewer. Studies rated as high quality (NOS ≥7) were included in the sensitivity analyses.

Data synthesis

When at least two studies reported comparable outcomes, a meta-analysis was conducted and reported HRs for OS, and their corresponding 95% CIs were extracted or calculated for each study. The HRs were log-transformed, and their standard errors were derived from the reported CIs.

Given the expected clinical and methodological heterogeneity among the included observational studies, we used a random-effects model with a restricted maximum likelihood (REML) estimator. To improve the accuracy of uncertainty estimates with the small number of included studies (k=4), we applied the Hartung-Knapp-Sidik-Jonkman adjustment to the random-effects meta-analysis. We interpreted heterogeneity qualitatively using I² and the magnitude and direction of study effects, acknowledging that I² is descriptive and should not be treated as a rigid categorical measure. Because of the limited number of studies, no subgroup analyses were conducted. Instead, we performed leave-one-out sensitivity analyses to examine the robustness of the pooled effect. We selected a random-effects framework because the included studies varied in exercise assessment methods, treatment exposure, geographic populations, and baseline prognostic factors. The small number of studies also imposes limitations on precision and increases uncertainty in between-study variance estimates, which we explicitly acknowledge as a methodological constraint.

The possibility of publication bias was evaluated visually using funnel plots and statistically using Egger’s regression test. In addition, sensitivity analyses excluding studies of low quality (NOS <7) were performed to assess the robustness of the pooled estimates.

All statistical analyses were conducted using standard meta-analysis procedures, with the pooled HR and 95% CI representing the estimated overall effect of exercise on survival in patients with mCRC.

All statistical analyses were performed using the latest version of EZR (Saitama Medical Center, Jichi Medical University, Saitama, Japan), a graphical user interface for R and R Commander that provides an integrated environment for conducting medical statistics and meta-analyses.

Results

Study Selection

**Figure 1:**
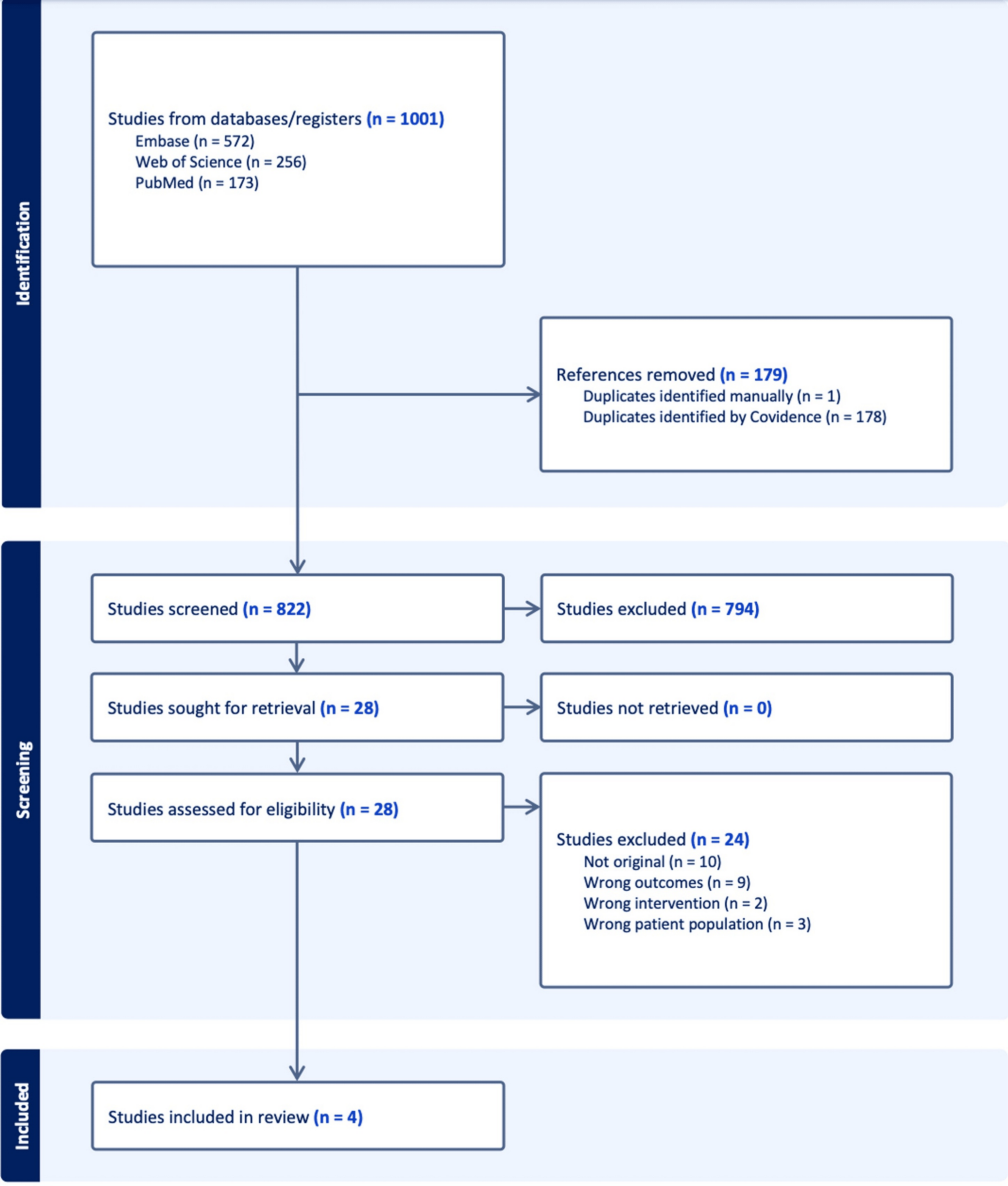
Selection flow

A total of 1001 records were identified through the systematic database search (Embase, n=572; Web of Science, n=256; PubMed, n=173). After removal of 179 duplicate records (178 identified by Covidence and one identified manually), 822 studies remained for title and abstract screening. Following initial screening, 794 records were excluded because they did not meet the eligibility criteria based on title or abstract review. The remaining 28 studies were sought for retrieval, and all were successfully obtained in full text. After full-text assessment, 24 articles were excluded for the following reasons: not original research (n=10), inappropriate outcomes (n=9), irrelevant interventions (n=2), and non-eligible patient populations (n=3). Finally, four studies met the inclusion criteria and were included in the quantitative synthesis. The complete study selection process is illustrated in Figure 1 (PRISMA 2020 flow diagram), which provides a detailed overview of the identification, screening, eligibility, and inclusion phases (Figure 1).

Characteristics of the Included Articles

Four prospective studies published between 2017 and 2022 were included in this review, all examining the association between physical activity and clinical outcomes in patients with mCRC. The studies were conducted in Germany, the United States, the Netherlands, and China, with sample sizes ranging from 40 to 1218 participants. All employed either prospective cohort designs or secondary analyses of randomized controlled trials.

Across studies, participants’ median ages ranged from 59 to 69 years, and men represented 59%-72% of the cohorts. Most studies adjusted for multiple confounding variables, including age, sex, body mass index (BMI), tumor site, treatment regimen, and performance status, to minimize bias.

Physical activity was assessed using validated self-administered questionnaires, including the International Physical Activity Questionnaire (IPAQ), the Short Questionnaire to Assess Health-Enhancing Physical Activity (SQUASH) questionnaire, and the CALGB physical activity instrument. Activity levels were generally expressed as metabolic equivalent tasks (METs) or categorized into low, moderate, and high activity groups based on leisure-time physical activity. The exercise content primarily involved aerobic activities, including walking, cycling, and treadmill exercise, conducted at moderate intensity (approximately 3-6 METs) and at a frequency of three to six sessions per week, for durations ranging from three to six months (Table 1).

**Table 1:** Demographic data of the included articles NR, not reported; PA, physical activity; IPAQ, International Physical Activity Questionnaire; SD, Standard deviation; SQUASH, Short Questionnaire to Assess Health-enhancing physical activity

Author	Year	Country	Study design	Sample size	Age	Male (%)	Exercise type	Assessment method
Walter V et al. [11]	2017	Germany	Prospective cohort	469	69 (30-96）	59	Walking	Interview-based recall survey
Guercio et al. [12]	2019	USA	Prospective cohort	1218	59 (46-69）	63	Aerobic or nonvigorous (walking, etc.）	Questionnaire
Smit et al. [13]	2022	Netherlands	Prospective cohort	293	62.9 (SD 10.6）	67.2	Combined (aerobic, resistance, and daily activity）	SQUASH questionnaire
Sun L et al. [14]	2022	China	Prospective observational follow-up	40	65 (SD 11)	72.5	Self-reported (walking, housework, and mild-to-moderate activity)	IPAQ

Results of meta-analysis

Overall Survival (OS)

Four studies reporting HRs for OS were included in the quantitative synthesis. Using a random-effects model (DerSimonian-Laird method), the pooled HR for OS among patients with mCRC who engaged in mild to moderate physical activity compared with those with no activity was 0.64 (95% CI 0.48-0.87), indicating a significant association between mild to moderate physical activity and improved survival.

**Figure 2:**
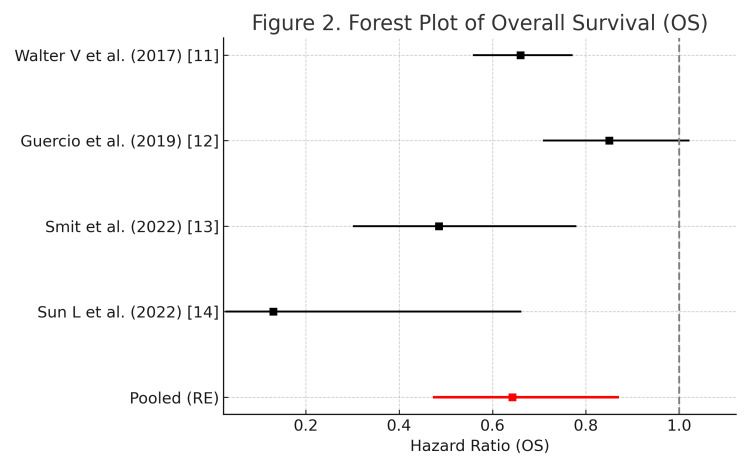
Forest plot of OS in patients with mCRC Forest plot of HRs for OS in patients with mCRC according to physical activity level. Each horizontal line represents an individual study’s HR and 95% CI for OS comparing higher versus lower (or no) physical activity. Values <1.0 indicate improved survival among patients with higher physical activity. The size of each marker indicates the relative weight of the study in the meta-analysis. The diamond at the bottom represents the pooled HR and 95% CI estimated using a random-effects model with a REML estimator and Hartung-Knapp-Sidik-Jonkman adjustment. The vertical dashed line indicates the null value (HR=1.0). Heterogeneity statistics (I², τ², and p-value for Cochran’s Q test) are shown in the figure to describe between-study variability. The forest plot was generated using the latest version of EZR (Saitama Medical Center, Jichi Medical University), a graphical user interface for R. The four included articles were Walter V et al. (2017) [11], Guercio et al. (2019) [12], Smit et al. (2022) [13], and Sun L et al. (2022) [14]. OS, overall survival; HR, hazard ratio; mCRC, metastatic colorectal cancer; CI, confidence interval; REML, restricted maximum likelihood

Substantial heterogeneity was observed among the included studies (I²=74.5%, τ²=0.055), suggesting variability in study design, physical activity assessment methods, and participant characteristics. The corresponding fixed-effects estimate was HR=0.71 (95% CI 0.63-0.80), consistent in direction but slightly attenuated. A leave-one-out sensitivity analysis confirmed the robustness of the pooled estimate, as exclusion of any single study did not materially change the results (HR range 0.61-0.69) (Figure 2).

Quality assessment 

The methodological quality of the included studies was appraised using the NOS for cohort studies. All four studies achieved scores ranging from seven to eight, indicating moderate to high methodological quality. All studies clearly defined their cohorts, inclusion criteria, and methods for assessing exposure (physical activity). Most were adjusted for key confounding variables, including age, sex, BMI, tumor site, treatment regimen, and performance status. Outcome ascertainment (OS) was complete in all studies, with follow-up durations of 18 to 74 months.

Potential limitations included the reliance on self-reported questionnaires to measure physical activity and incomplete reporting of changes in physical activity over time. Additionally, the absence of randomization and variation in the timing of physical activity assessment may have introduced residual confounding and measurement bias (Table 2).

**Table 2:** Quality assessment of the included studies using the NOS Each study was evaluated according to three domains: selection (maximum 4 points), comparability (maximum 2 points), and outcome (maximum 3 points). The total score ranges from 0 to 9, with higher scores indicating better methodological quality. Studies scoring 7 to 9 were considered high quality, 5 to 6 as moderate quality, and <5 as low quality. NOS: Newcastle-Ottawa Scale

Author	Year	Selection (max 4)	Comparability (max 2)	Outcome (max 3)	Total NOS score	Quality rating
Walter V et al. [11]	2017	4	2	2	8	High
Guercio et al. [12]	2019	4	2	2	8	High
Smit et al. [13]	2022	4	2	2	8	High
Sun L et al. [14]	2022	3	2	2	7	Moderate

Publication bias

**Figure 3:**
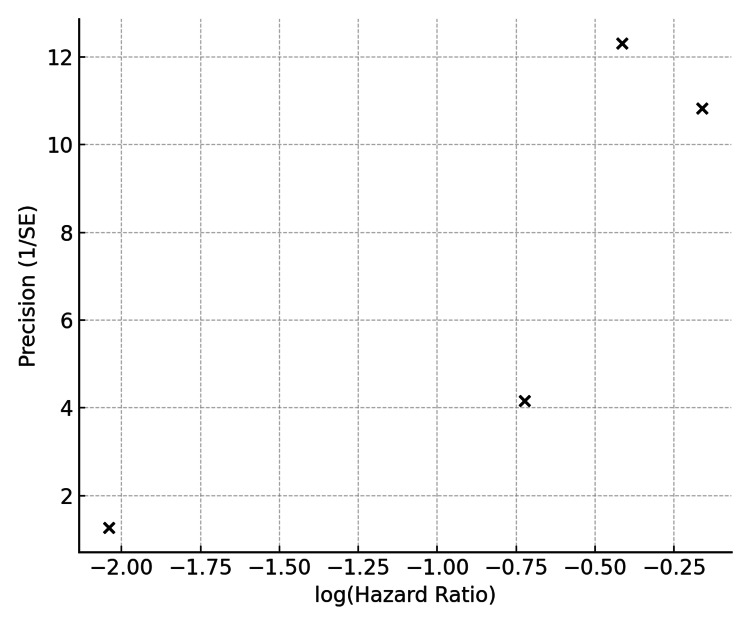
Funnel plot for publication bias Funnel plot evaluating potential publication bias and small-study effects in the four included studies reporting HRs for OS. Each point represents an individual study plotted by the natural logarithm of the HR (log[HR]) on the x-axis and the SE on the y-axis, with smaller SEs (more precise studies) appearing toward the top of the plot. Visual asymmetry may indicate the presence of small-study effects, selective reporting, or publication bias. The funnel plot was generated using the latest version of EZR (Saitama Medical Center, Jichi Medical University), a graphical user interface for R. Given the small number of studies (k=4), formal statistical tests of funnel plot asymmetry (e.g., Egger’s regression or Begg’s test) were not performed, as these tests are unreliable when k<10. The pooled effect shown in the plot was estimated using a random-effects model with a REML estimator and Hartung-Knapp-Sidik-Jonkman adjustment. HR, hazard ratio; SE, standard error; REML, restricted maximum likelihood; OS: overall survival

Visual inspection of the funnel plot for OS suggested mild asymmetry, with smaller studies tending to report more substantial beneficial effects of physical activity on survival. Given the limited number of included studies (n=4), statistical testing for funnel plot asymmetry (e.g., Egger’s or Begg’s test) was not performed, as such tests lack reliability when fewer than 10 studies are included (Figure 3).

Discussion

Summary of the Study

This systematic review and meta-analysis demonstrated that mild to moderate physical activity was significantly associated with improved OS among patients with mCRC. The pooled HR for OS was 0.64 (95% CI: 0.48-0.87) using a random-effects model, indicating a 36% reduction in mortality risk among physically active patients compared with those who were less active. Although heterogeneity was substantial (I²=74.5%, τ²=0.055), the direction of effect was consistent across all studies. However, the feasibility of engaging in even low-intensity activity varies significantly across the mCRC population and should not be viewed as universal. Patients with progressing disease, significant liver involvement, pronounced cachexia, chemotherapy-induced fatigue, or peripheral neuropathy may be unable to maintain even light daily movement, and activity levels can fluctuate markedly over short intervals as performance status changes.

The included studies mainly assessed non-vigorous activities, such as walking, household chores, and light shopping, suggesting that even modest daily movement may yield survival benefits for patients with advanced disease. Given that mCRC patients often experience chemotherapy-induced fatigue, neuropathy, and general frailty, mild to moderate activities may represent a realistic and safe level of exercise [15]. Importantly, many of these patients also live with multimorbidity, emphasizing the need for collaboration between oncologists and general physicians to support the integrated management of comorbidities and to encourage sustainable, patient-tailored exercise routines [16,17].

Comparison With Other Studies

Previous research on exercise and survival in CRC has primarily focused on non-metastatic or locally advanced disease, with most trials assessing moderate-to-vigorous aerobic programs [18-20]. These studies often reported HRs ranging from 0.55 to 0.75 for high versus low activity, consistent with the direction observed in our meta-analysis [18-20]. However, HRs from early-stage cohorts cannot be considered biologically or clinically comparable to mCRC, because the prognostic architecture changes profoundly once systemic disease is present. Patients with mCRC experience rapid shifts in functional capacity due to treatment fatigue, sarcopenia, cachexia, neuropathy, and cumulative toxicity, all of which directly limit exercise tolerance and alter the potential physiological responsiveness to physical activity.

Some reports suggested that vigorous or structured exercise confers greater benefits, while others found no additional advantage over moderate activity [21,22]. Our findings refine this understanding by showing that even mild to moderate intensity exercise, often feasible for patients with advanced disease, may still be associated with improved OS. Although this association cannot be interpreted as causal due to the observational nature of included studies, the pooled estimate and sensitivity analysis (leave-one-out range HR=0.61-0.69) support the robustness of the association.

Strengths of the Study

To our knowledge, this is the first meta-analysis to specifically quantify the survival impact of mild to moderate physical activity in mCRC. The focus on realistic, low-intensity exercise provides practical insights for clinical oncology and survivorship care [23-25]. Unlike prior analyses that emphasize high-intensity regimens, this study highlights achievable and safe activity levels that can be integrated into patients’ daily routines, even during active chemotherapy.

Methodologically, this review adhered to PRISMA 2020 guidelines and included only prospective designs with adjusted HRs for OS. Study quality evaluated using the NOS ranged from seven to eight points, indicating moderate to high methodological rigor. The use of random-effects modeling and sensitivity analyses further strengthened the reliability of the findings.

Limitations

Several limitations must be considered when interpreting the results of this meta-analysis. The evidence base was small (k=4), which precludes any meaningful assessment of publication bias; therefore, funnel plot asymmetry should not be interpreted, and no conclusions about selective reporting can be drawn. The included studies relied on self-reported physical activity, which is particularly problematic in mCRC because fatigue, neuropathy, cachexia, and fluctuating performance status can impair accuracy and lead to systematic overestimation of actual activity.

Heterogeneity across studies was substantial and reflects clinically essential sources of variability, including differences in disease burden (e.g., liver-dominant versus limited metastatic spread), treatment intensity and toxicity profiles, molecular subtype, and the timing of activity measurement (pre-treatment, during chemotherapy, or after progression). These factors strongly influence functional capacity and prognosis, and inconsistent adjustment for them complicates the interpretation of pooled estimates.

Although residual confounding was acknowledged, its impact is likely profound. Reverse causation is a central concern: patients with greater functional reserve, more favorable tumor biology, less treatment toxicity, and better tolerance of systemic therapy are inherently more able to engage in physical activity and also more likely to survive longer. As a result, the association observed in this meta-analysis may primarily reflect baseline prognostic advantage rather than any independent effect of physical activity on survival.

Moreover, data on adherence, temporal changes in activity, and dose-response relationships were sparse, preventing meaningful evaluation of whether higher levels of activity or sustained engagement influence outcomes. Together, these limitations underscore that the findings should be interpreted cautiously and viewed as hypothesis-generating, rather than establishing a causal or clinically actionable relationship between physical activity and survival in mCRC.

## Conclusions

This systematic review and meta-analysis suggest that higher levels of mild to moderate physical activity may be associated with improved OS in patients with mCRC. However, given the small number of available studies, reliance on self-reported activity, substantial heterogeneity, and the strong likelihood of residual confounding and reverse causation, these findings should be interpreted with considerable caution. In mCRC, physical activity is closely intertwined with evolving performance status, treatment tolerance, symptom burden, and disease trajectory; the observed associations may therefore reflect underlying functional reserve rather than a direct physiological effect of activity on survival. Accordingly, the present results are best viewed as hypothesis-generating rather than evidence of a causal or clinically actionable relationship. To clarify whether physical activity exerts an independent prognostic influence in mCRC, future research should incorporate standardized, objective, and time-dependent measures of physical activity, such as accelerometry, as well as rigorous adjustment for disease burden, molecular subtype, systemic therapy, inflammatory status, and functional decline. Prospective studies with repeated assessments across the treatment continuum will be essential to understanding whether physical activity can meaningfully inform supportive care strategies or prognostic evaluation in this population.
